# Bottom sediments as an indicator of the restoration potential of lakes—a case study of a small, shallow lake under significant tourism pressure

**DOI:** 10.1038/s41598-024-64058-9

**Published:** 2024-06-11

**Authors:** Katarzyna Kowalczewska-Madura, Julita A. Dunalska, Sebastian Kutyła, Szymon Kobus

**Affiliations:** 1 Department of Water Protection, Faculty of Biology, Adam Mickiewicz University, Uniwersytetu Poznańskiego 6, 61-614 Poznań, Poland; 2 Faculty of Oceanography and Geography, Center for Water Monitoring and Protection, University of Gdańsk, Jana Bażyńskiego 8, 80-309 Gdańsk, Poland; 3 Department of Freshwater Protection, Institute of Environmental Protection – National Research Institute, Słowicza 32, 02-170 Warsaw, Poland; 4 Department of Water Management and Climatology, Faculty of Agriculture and Forestry, University of Warmia and Mazury in Olsztyn, Plac Łódzki 2, 10-708 Olsztyn, Poland

**Keywords:** Bottom sediments, Indicator, Internal loading, Lake restoration, Tourism pressure, Biogeochemistry, Environmental sciences, Limnology

## Abstract

The study covered a small, shallow lake, intensively used for recreation (sailing, tourist services and port infrastructure). This study aimed to determine the spatial differentiation of bottom sediments and the potential for phosphorus release in five zones, differing mainly in the type of recreation, depth, direct catchment management, shoreline management and macrophyte presence. The results were used to propose protective and restoration measures to improve the water quality of the studied lake. The innovation in the study was the detailed analysis of bottom sediments, which can be a significant source of pollution besides the external load from the catchment and tourist pressure, in the planned management of this ecosystem. Examination of the physicochemical properties of the bottom sediments showed a clear variation in both composition and potential for internal phosphorus loading. The sediments from the profundal zone, where the most boating activity was observed, together with the sediments from the shallow zone where the boats dock (mooring zone), had the highest potential to supply phosphorus to the bottom waters. This fact was demonstrated by the highest total phosphorus (TP) concentrations in sediments (up to 1.32 mgPg^−1^ DW) and the content of the most mobile fractions (up to 33%). The other zones associated with the marina, fuel zone, tributary and canal were not significant sources of phosphorus to the ecosystem. Based on the above results, a restoration method involving the removal of bottom sediments from the bottom zone was proposed, supported, of course, by protective measures in the catchment (maintaining a buffer zone around the lake and limiting the inflow of pollutants with tributary waters). The proposed measures with sustainable tourist pressure should improve water quality and thus contribute to protecting this valuable natural landscape.

## Introduction

Lakes are particularly important freshwater habitats significantly attracting the public. Many anthropogenic impacts are linked to the construction of infrastructure such as tourism facilities, including resorts, hotels, restaurants and marinas^1^. Tourism impacts lakes usually through several recreational activities. The most important ones are swimming, boating and angling. The negative impacts of tourism development can gradually destroy the environmental resources. Tourist activity can be an important source of nutrients directly or indirectly entering surface waters. The natural values of areas located near lakes promote the destruction of shorelines by recreational development, construction of piers, removal of vegetation to create wild beaches and bathing beaches, and littering of shores^2–4^. Boats directly and indirectly affect many lakes, littoral and shoreline water parameters. Extensive operation of power boats can have a variety of impacts on water bodies: sediment resuspension, water pollution, fish and wildlife disturbance, aquatic plant destruction and shoreline erosion^5,6^. Boat wakes erode shorelines, scour the bottom of the shoreface, and decrease water clarity through turbulence^7^.

Water quality in lakes depends on processes in the catchment area (external loading) and in the aquatic ecosystem (internal loading). Bottom sediments in lakes are an important part of the ecosystem because they act as temporary or permanent sinks for various compounds. Natural sediments are a mixture of different compositional types: eroded original rock material, clay minerals, precipitates and coatings and organic matter. They can also include heavy metals, biogenic compounds and other pollutants^8–10^. If the sediments are contaminated, the concentrations of harmful substances in water and sediments and the potential ecosystem effects related to such concentrations increase^11^. Phosphorus accumulated in sediments for many years is subsequently often released into the water column. Lake sediments become a source of internal phosphorus loading, maintaining a high trophic state and bad water quality^12–14^. The amount of phosphorus released from sediments into the overlying water depends on temperature pH, oxygen concentration in overlying water, redox conditions, resuspension processes and sediment structure^10,15^. The potential for internal loading of phosphorus also results from the presence of a particular fraction of phosphorus in the sediment and the possibility of conditions promoting its release into the water. A fractionation scheme determines the fractions of phosphorus bound to metals and organic matter. Analysis of the ratios of fractions with different bioavailability is a very important source of information about the persistent phosphorus accumulation in sediments and the potential for phosphorus release into the water body. When assessing bottom sediments as a potential source of phosphorus, its bioavailability may be more important than its total content in the sediment^16–18^.

The importance of the foregoing factors strongly depends on the morphometry, especially on the depth of the water body, macrophyte presence and the catchment area uses^16,19^. The major factor responsible for the phosphorus load released from bottom sediments is the trophic state of the lake. In the water layer above the bottom, the anaerobic conditions observed cause a very low redox potential, which promotes the release of phosphorus accumulated on metal compounds, especially iron oxyhydroxides^15^. The chemical composition of the bottom sediment in lakes reflects the type and intensity of the anthropogenic influence on the lake and its catchments and provides information on potential threats to the ecosystem^20^. Knowledge of phosphorus concentration in the interstitial and overlying water may help in predicting the phosphorus amount potentially available in the process of internal loading from bottom sediments^21^. This phenomenon is especially important for lakes susceptible to significant nutrient inputs from the catchment, where sediments can provide an additional source of supply and contribute to the deterioration of water quality.

Shallow lakes are particularly susceptible to eutrophication and are vulnerable to internal loading because biogenic substances accumulated in their sediments have a greater impact on water quality than in deeper lakes^22,23^. In shallow, polymictic lakes are observed rapid, short-term changes of aerobic conditions in the overlying water, short-lived stratification, turbulent mixing and an increased water flow^24^. In lakes of shallow depth, even low wind velocities can considerably resuspend the deposits on the lake bed^11^. Boats also intensify resuspension in shallow lakes^6^. In such systems, internal phosphorus loading from bottom sediments often represents the main summer phosphorus load, which strongly influences primary production and algal bioavailability^25,26^. In shallow non-stratified lakes, in which the water column remains oxic throughout the growing season, internal phosphorus loading can also occur through various mechanisms, i.e.: sediment resuspension due to waves, water currents, biota, mineralisation of organic matter and diffusion^27,28^.

To improve the water quality of a eutrophic lake, all external sources of nutrients flowing in from the catchment area should first be cut off (catchment protection measures). However, with lakes in which bottom sediments are a significant source of phosphorus (pollution loads accumulated in the period before introducing protective measures), the above measures may be insufficient. Implementing suitable restoration methods in the lake itself is then necessary, which significantly reduces the phosphorus returning from the bottom sediments to the hypolimnion waters and further to the epilimnion, contributing to the high primary production of cyanobacterial blooms. The methods for lake restoration can be divided into physical (removal of bottom sediments, flushing of lakes, drainage of hypolimnion waters and destratification of lakes), chemical (inactivation of phosphorus using coagulants, e.g. aluminium, iron or calcium salts; oxygenation of hypolimnion waters and dosage of nitrate) and biological (biomanipulation, introduction of effective microorganisms and macrophytes and application of barley straw)^29–31^. The selection of an appropriate restoration method for a lake should be followed by a detailed study of the aquatic ecosystem, including both water and bottom sediments. Often, combining several complementary restoration methods, e.g. physical and biological, offers the best results^32,33^.

The Sztynorckie Lake is an example of a heavily polluted, shallow lake under significant recreational and catchment area pressure. The threats to water quality from both recreational use (marina, sailing tourism and boat refuelling station) and catchment area loads (tributaries, surface runoff from meliorated and agriculture areas) cause its waters to be highly eutrophic. Pressures primarily from the different uses of the lake have caused the bottom sediments of this small lake to have a differentiated character. We hypothesise that the bottom sediments of this small, shallow lake are differentiated in their ability to release phosphorus into the overlying waters, depending on the type of pressure. Moreover, they act as an additional source of this element, contributing to the deterioration of the trophic state of the waters. Sztynorckie Lake and its bottom sediments may represent a model system formed by lake zones with different impacts of the management of the reservoir itself and its catchment.

The aim of the study was to determine the differentiation of the bottom sediments of Sztynorckie Lake and primarily, to assess the possibility of phosphorus release from bottom sediments as a source of internal loading. In the lake, five zones have been designated, differing mainly in the intensity of recreation activity (especially boating), use of the direct catchment area (tourism infrastructure/agriculture areas), depth of water and presence of macrophytes. Based on the results of the study, restoration methods have been proposed for this highly eutrophic lake, which will aim to improve water quality by reducing the internal source of nutrients while maintaining a sustainable approach to the recreational use of the lake.

## Materials and methods

### Study lake

#### Morphological and hydrological parameters

**Figure 1:**
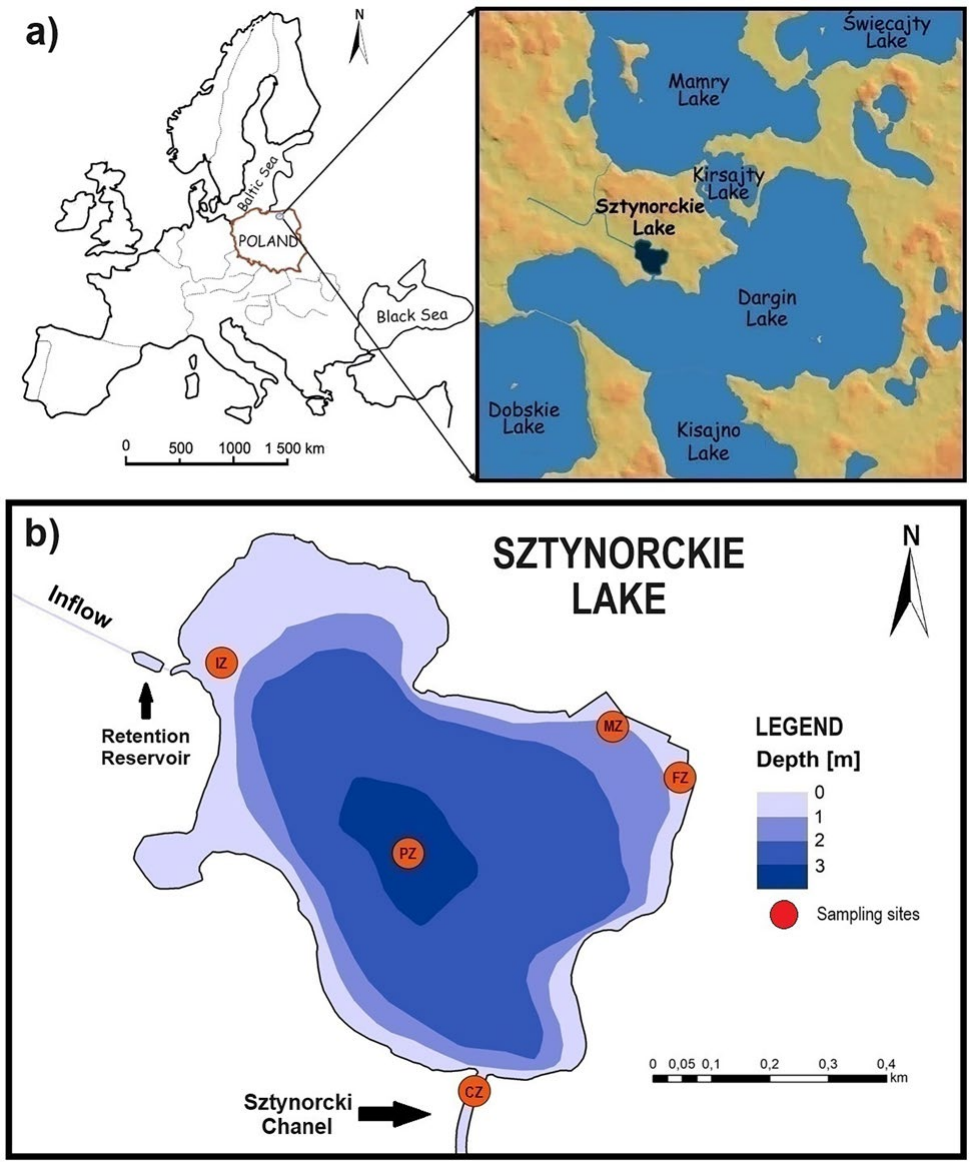
The location (**a**), bathymetric map and position of sampling stations in Sztynorckie Lake (**b**) [PZ—Profundal Zone (station 4), MZ—Mooring Zone (station 5), FZ—Fuel Zone (station 3), IZ—Inflow Zone (station 1), CZ—Canal Zone (station 2)] (own elaboration based on IFI^43^ and Greala et al.^44^).

Sztynorckie Lake is located in the Great Masurian Lake District, north-eastern Poland (54° 7′ 42.187″ N, 21° 40′ 40.44″ E; Fig. 1a)^34^. The whole Masurian Lakeland is one of the most important recreational sites in Poland. Therefore, the lake system is greatly affected by tourism, which has been developing. In the past few years, the number of tourists has increased significantly^35–38^. Growing anthropopressure over the last several decades has rapidly and progressively eutrophied the Great Masurian Lakes system^38,39^. Sztynorckie Lake is a 50.2 ha (with an overgrowing bay in the eastern part) shallow, unstratified (polymictic) water body, with a maximum depth of 3.1 m and water exchange of 90% per year (Fig. 1b). The poorly developed shoreline (development index–1.43) is 3603 m long. The water volume of the lake is 906.9 ths. m^3^ (Table 1). According to the Lake Quality Assessment System developed by Kudelska et al.^40^, considering mainly morphometric and hydrological conditions, Lake Sztynorckie is very susceptible to degradation due to its mean depth (1.9 m), percent of stratifications (0%) and ratio of active bottom-to-lake volume (0.55). This lake is classified as a flow-through lake. The lake is recharged by water from a 4069 m long drainage ditch (drains nearby agricultural areas, water from the ditch periodically discharging to a retention reservoir). Water is drained from Sztynorckie Lake to Dargin Lake via an artificial Sztynorckie Canal, built in 1765–1772, and then modernised in 1854–1857^41^. Water in the canal is stagnant (no flow)^42^.

**Table 1:** Morphometric and hydrological characteristics of Sztynorckie Lake.

Parameter (unit)	Value	References
Area (ha)	50.2	Grela et al.^44^
Lenght of the shoreline (m)	3603	
Maximum length (m)	1035	
Maximum width (m)	865	
Volume (ths. m3)	906.9	Cydzik^45^
Maximum depth (m)	3,1	
Mean depth (m)	1,9	
Ratio of lake volume to length of the shoreline	168.4	
Ratio of active bottom to lake volume	0.55	
Percent of stratifications (%)	0	
Water exchange [% per year]	90	
Shoreline development index	1,43	Own elaboration based on IFI^43^ and Grela et al.^44^
Schindler’s ratio	9.2	

#### Water quality

The water quality of Sztynorckie Lake was examined in 2 years, 1997^42^ and 2020^46^. In both these periods, physicochemical parameters indicated strong eutrophication of the lake. In 2020, significantly lower values of total nitrogen and conductivity were recorded, accompanied by a large increase in chlorophyll a (Table 2). The other parameters remained at a similar level. The trophic state of the lake is on the boundary between eutrophy and hypertrophy^47^. In August 1997, the concentration of heavy metals in the water was additionally determined: lead (0.0195 mgPb L^−1^), copper (0.0027 mgCu L^−1^), zinc (0.023 mgZn L^−1^) and cadmium (mgCd L^−1^)^42^. Concentrations of heavy metals were not analysed in 2020.

**Table 2:** Physicochemical parameters in the surface layers of Lake Sztynorckie in August 1997^42^ and 2020^46^.

Parameter	Unit	August 1997	August 2020
Temperature	°C	22.7	21.2
Oxygen	mgO_2_ L^−1^	10.1	11.5
Conductivity	µS cm^−1^	392	300
pH	–	8.9	9,0
Total phosphorus	mgP L^−1^	0.08	0.08
Total nitrogen	mgN L^−1^	2.61	1.72
Chlorophyll a	µg L^−1^	60.3	96.1
Secchi disk visibility	m	0.4	0.5

#### Macrophytes

**Figure 2:**
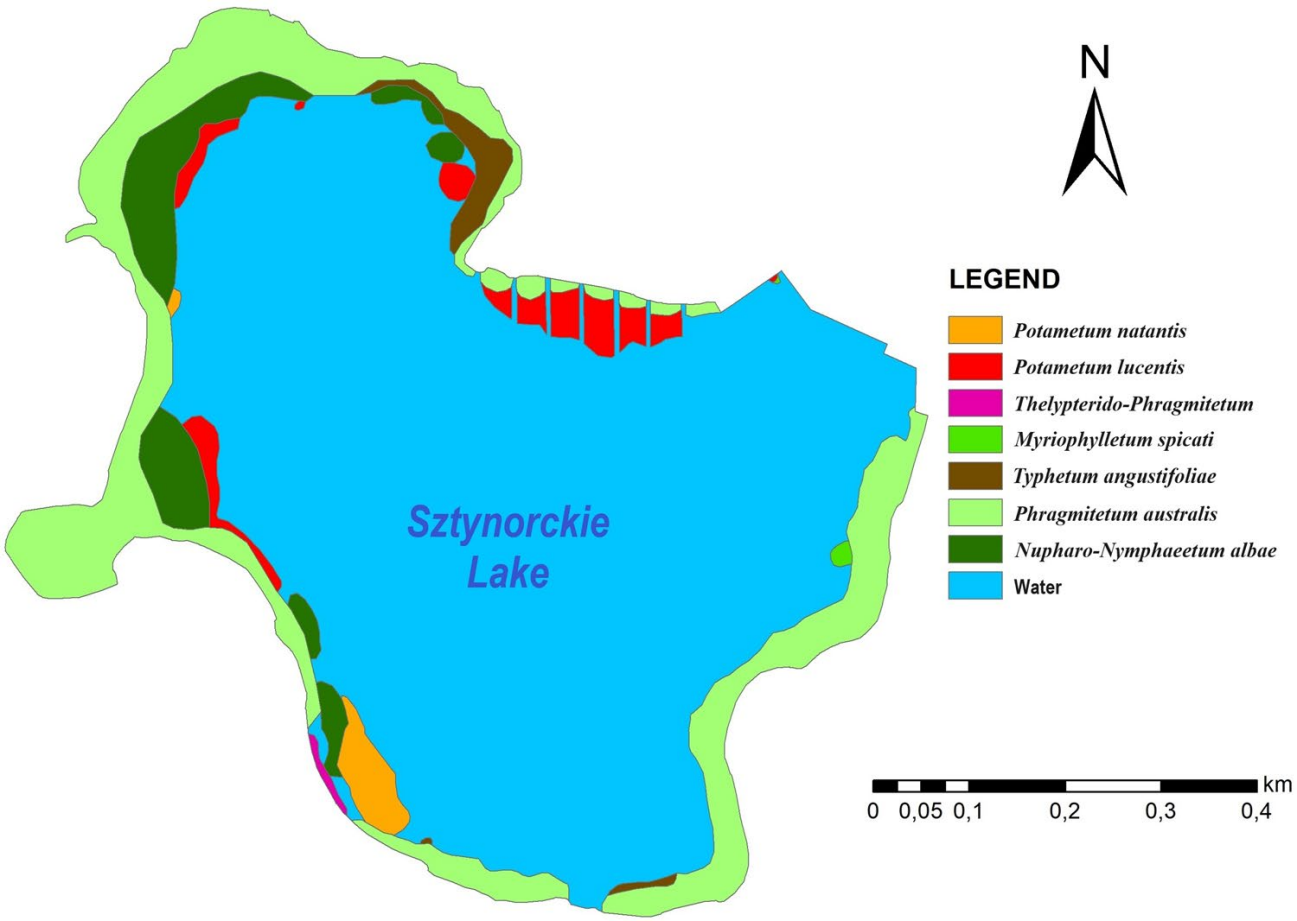
Distribution of macrophyte communities in Sztynorckie Lake during the growing season 2020 (after Ławniczak-Malińska^48^).

Based on the previous research^48^, the taxonomic diversity of macrophytes in Sztynorckie Lake was low, and macrophytes covered approximately 25% of the total lake area. The aquatic vegetation was composed of submerged, emergent and floating-leaf plant communities and included two communities of elodeids (*Potametum lucentis* Hueck 1931 and *Myriophylletum spicati* Soó 1927 ex Podbielkowski et Tomaszewicz 1978), two of nympheids (*Nupharo-Nymphaeetum albae* Tomasz. 1977 and *Potametum natantis* Soó 1923) and two of helophytes (Phragmitetum australis [Gams 1927] Schmale 1939 and *Typhetum angustifoliae* [Allorge 1922] Soó 1927). In the shore zone, a swamp community *Thelypterido-Phragmitetum* Kuiper 1958 has developed (Fig. 2).

#### Sources of pollution

Sztynorckie Lake was polluted with slurry and sewage from the farm in the 1970s–1980s, which entered through a drainage ditch (BZT_5_ = 100 mgO_2_ L^−1^, total dissolved solids 1124 mg L^−1^). An inspection of water and sewage management in 1990 showed the outflow of sanitary sewage directly into the lake in amounts of approximately 24 m^3^ day^−1^. The sewage reached from the sanitary facilities of the recreation centre and residential buildings in the village of Sztynort located on the shore of the lake. In 1991, sewage inflows were closed, and a mechanical–biological treatment plant is currently in operation^42^.

The pollution also enters Sztynorckie Lake through a tributary from the north-western side, which drains a small retention reservoir (flow 150 L s^−1^, Fig. 1b). It is characterised by significant electrolytic conductivity (up to 854 µs cm^−1^) and high concentrations of total nitrogen (up to 10.1 mgN L^−1^) and total phosphorus (0.19 mgP L^−1^). Additionally, high counts of coliforms and *E. coli* were found in its waters^49^.

The Sztynorckie Lake is intensively used for recreation and has a yacht port, which can operate 600 boats. The equipment in the marina area allows for full yachting services, including necessary repairs. Open throughout the year, the highest number of boats in the port is observed between the beginning of May and the end of September. In summer, the number of sailors on the lake reaches 16,000 per month (Sztynorckie Port data). The marina offers a restaurant and a water equipment rental service. Sailors also have several resting places on the shore.

#### Catchment land use/cover

**Figure 3:**
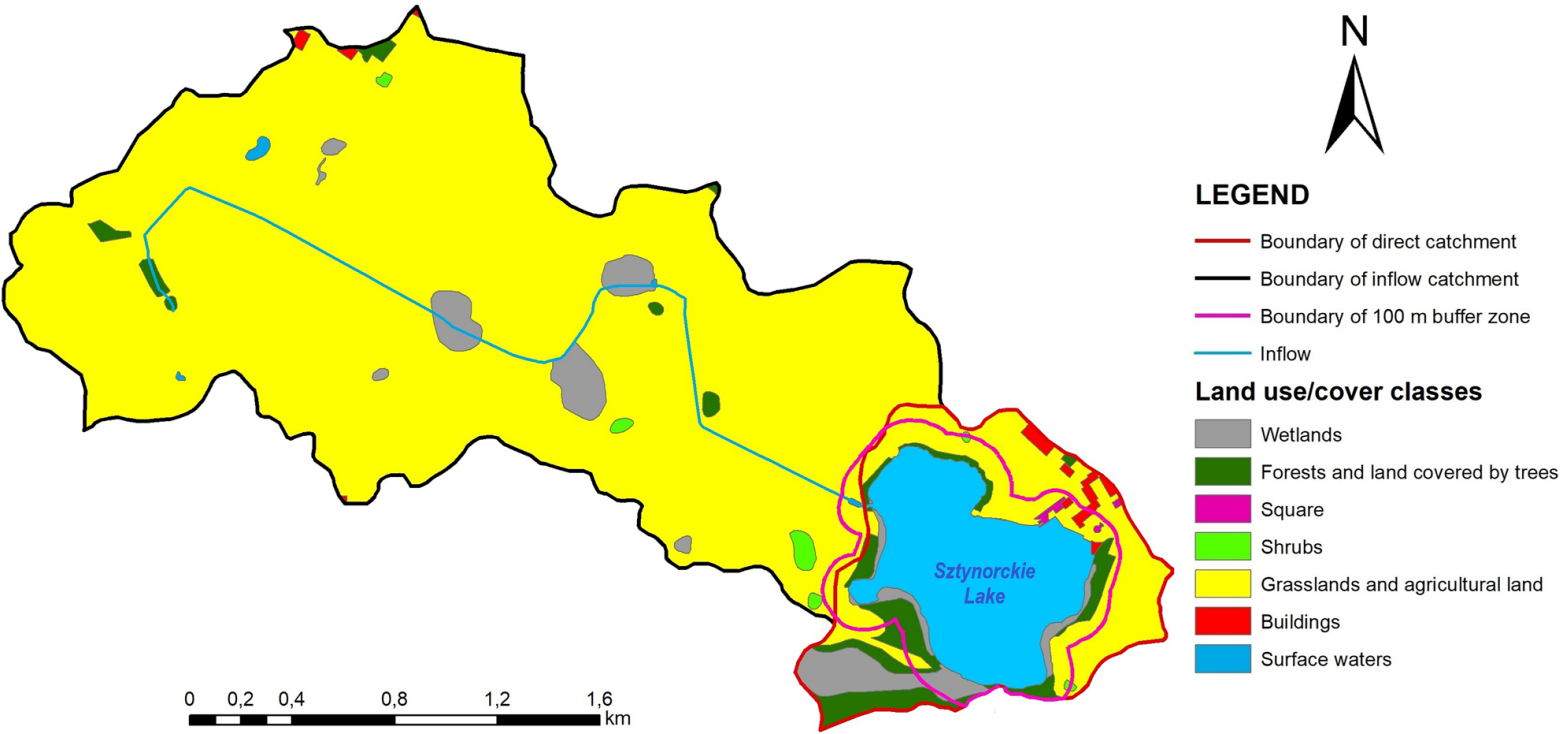
The land use differentiation of the catchment area of Sztynorckie Lake (own elaboration based on MIA^50^).

The total catchment area of Sztynorckie Lake amounts to 510.43 ha, including that of the inflow at 440.44 ha and the direct catchment area at 69.99 ha. The analysis of land use in the catchment area, conducted based on the Database of Topographic Objects at a scale of 1:10,000^50^, showed that over 95% of the inflow catchment area consists of grasslands and agricultural land. In the case of the direct catchment area and the 100-m buffer around the lake, the situation is better. In these two areas, over 20% are occupied by forests and land covered by trees, and wetlands account for about 15%. Nevertheless, grasslands and agricultural land in these two areas cover approximately 58% (Table 3, Fig. 3). According to the theoretical nutrient load generated from the lake catchment area, proposed by Pasztaleniec and Kutyła^51^, 3062.8 kgN and 126.2 kgP enter Sztynorckie Lake from the total catchment per 1 year.

**Table 3:** Land use/cover of Sztynorckie Lake catchment (own elaboration based on MIA^50^).

Land use/cover classes	100 m buffer zone		Direct catchment		Inflow catchment	
	ha	%	ha	%	ha	%
Wetlands	4.91	13.95	11.15	15.93	11.51	2.61
Forests and land covered by trees	8.62	24.53	15.15	21.65	3.54	0.80
Square	0.44	1.24	0.54	0.77	0.00	0.00
Shrubs	0.16	0.44	0.26	0.37	2.00	0.45
Grasslands and agricultural land	20.38	57.98	40.70	58.15	420.50	95.49
Buildings	0.55	1.58	2.19	3.13	2.19	0.51
Surface water	0.10	0.28	–	–	0.60	0.14

#### Climatic conditions

The climatic conditions of the Great Masurian Lake District are related to the clash of marine and continental climatic influences in the area, as well as to local factors (the large area of forests and the presence of lakes). They are characterised by strong seasonality (warm summers and cold winters)^52^. The average annual air temperature is about 7–8 °C. The lowest average monthly temperatures occur in January (from − 3 to − 2.5 °C) and the highest in July (18–18.5 °C). Annual precipitation ranges from 600 to 650 mm, reaching its highest values in June and lowest in February. Snow cover is present for about 65 days, and its thickness is about 11–12 cm^53^. Average monthly wind speeds exceed 3 m s^−1^ ^52^. The vegetation period in the analysed area lasts approximately 225 days^53^.

### Sampling and physicochemical analysis of bottom sediments and overlying water

Samples of bottom sediments from Sztynorckie Lake were taken once in August 2020 at five sampling stations characterising individual zones (Fig. 1b) related to different recreation uses of the lake and its catchment area. Station 1 (54° 07′ 51″ N, 21° 40′ 16″ E) was situated in the canal between the pumping station and Sztynorckie Lake (outflow from the retention reservoir located before the lake) and represented the Inflow Zone (IZ) influenced by the agriculture catchment area. Station 2 (54° 07′ 27″ N, 21° 40′ 42″ E) was in the Sztynorcki Canal, connecting Sztynorckie Lake with Dargin Lake (Canal Zone–CZ), intensively used by boating. The other sampling stations were located in Sztynorckie Lake: station 3 (54° 07′ 46’’ N, 21° 41′ 02″ E) near the Fuel Zone (FZ; transformation of shoreline), station 4 (54° 07′ 43″ N, 21° 40′ 41″ E) in the deepest place in the lake (Profundal Zone-PZ) and station 5 (54° 07′ 50″ N, 21° 40′ 38″ E) in the port, in the sailboat Mooring Zone (MZ, tourist service and port infrastructure).

Bottom sediments were sampled from 0 to 5 cm and 5–10 cm at each station, using a Kajak tube sampler^54,55^. For analysing total phosphorus (TP) and fraction content, phosphorus was fractionated in the collected sediment samples, using the protocol proposed by Psenner et al.^56^ and Lewandowski et al.^57^. In a volume of 1 cm^3^ of wet sediment, the following were analysed: loosely adsorbed phosphorus (NH_4_Cl-P)—extraction with 1 M NH_4_Cl; redox-sensitive phosphorus bound with iron (BD-P)—extraction with a mixture (1:1) of 0.11 M NaHCO_3_ and 0.11 M Na_2_S_2_O_4_; phosphorus bound to hydrated oxides of aluminium (NaOH-P) and organic matter (NaOH-NRP)—extraction with 1.0 M NaOH; carbonate- and apatite-bound P (HCl-P)—extraction with 0.5 M HCl. After each extraction, the sediment sample was centrifuged at 3000 rpm for 20 min, and the phosphorus content of the resulting solution was determined by the molybdate method with ascorbic acid as a reducer (PN-EN ISO 6878:2006^58^). The residue (Res-P) was the difference between TP concentration and the sum of the first five fractions. Total phosphorus content in the bottom sediments was analysed after incineration at 550 °C and extraction with hydrochloric acid (1:1), using the molybdate method with ascorbic acid as a reducer.

Sediment samples were also analysed for organic matter content (%) by drying to constant weight and incineration at 550 °C. Water content (%) was calculated from a difference between the wet and dry weight of the sample.

Pore waters from the bottom sediment were separated by centrifugation for 1 h at 3000 revolutions per minute in closed containers. Soluble reactive phosphorus (SRP) concentration was analysed in the supernatant with the molybdate method with ascorbic acid as a reducer, and total phosphorus (TP) with the same method, after mineralisation.

In the overlying water at the Profundal Zone, the following parameters were measured from April 2020 to October 2020 (at 2-month intervals): water temperature, dissolved oxygen concentration, electrolytic conductivity and pH using a YSI Professional Plus meter. SRP and TP concentrations were also analysed in the water layer above the sediment at five sampling stations.

## Results

### Physicochemical parameters of bottom sediments

The water content in the sediments in Sztynorckie Lake ranged from 29.8 to 95%. The minimum was observed in FZ in the 5–10 cm layer and the maximum in PZ in the 0–5 cm layer. Higher values of this parameter (over 70%) were noted at sampling stations located in PZ and MZ (Table 4). Dry residue reached the highest values in FZ—702 g kg^−1^ WW. Significantly lower values were observed in PZ and MZ with the minimum in PZ—50.1 g kg^−1^ WW. Except for zone CZ, a higher content of this parameter was noted in the 5–10 cm layer (Table 4).

**Table 4:** Water content, dry residue, total phosphorus and organic matter in bottom sediments in Sztynorckie Lake in five zones in two layers (0–5 cm and 5–10 cm) [abbreviations—see Fig. 1].

Zone		Water content (%)	Dry residue (g kg^−1^WW)	Total phosphorus (mgP g^−1^ DW)	Organic matter (%)
IZ	0–5 cm	69.1	308.9	0.71	15.2
	5–10 cm	55.0	450.1	0.66	10.2
CZ	0–5 cm	31.2	687.7	0.37	22.1
	5–10 cm	53.9	461.2	0.38	17.2
FZ	0–5 cm	35.8	642.1	0.28	4.6
	5–10 cm	29.8	702.1	0.27	3.1
PZ	0–5 cm	95.0	50.1	1.26	33.3
	5–10 cm	91.3	87.4	1.32	31.3
MZ	0–5 cm	87.0	130.3	0.68	22.7
	5–10 cm	78.5	214.9	0.84	16.7

The concentration of total phosphorus in bottom sediments in Sztynorckie Lake varied in a wide range between 0.27 mgP g^−1^ DW (FZ, in layer 5–10 cm) and 1.32 mgP g^−1^ DW (PZ, in layer 5–10 cm). In both canal zones (IZ and CZ) it was lower and ranged from 0.37 to 0.71 mgP g^−1^ DW. The content of this element was similar in both analyzed layers. The mean TP concentration in lake sediments was slightly higher at 0.77 mgP g^−1^ DW than in canals − 0.53 mgP g^−1^ DW (Table 4).

**Figure 4:**
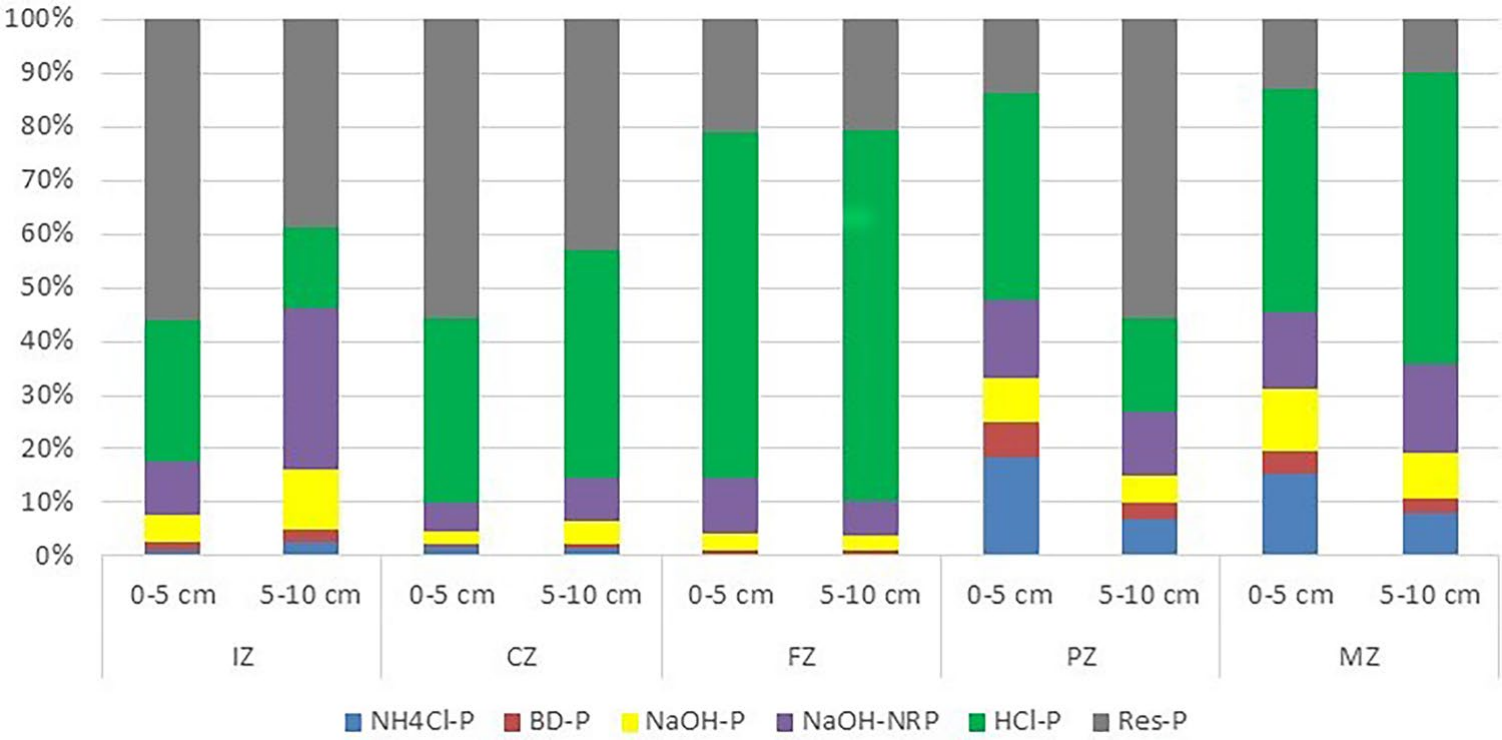
The percentage share of extractable fractions of total phosphorus in the bottom sediments of Sztynorckie Lake in five zones in two layers (0–5 cm and 5–10 cm) [abbreviations—see Fig. 1].

The share of particular fractions in total phosphorus in bottom sediments of Sztynorckie Lake was spatially quite differentiated. In IZ the dominant phosphorus fraction in both layers was Res-P (56% and 39% respectively) i.e. practically unavailable phosphorus. The sum of fractions with the highest bioavailability (NH_4_Cl-P, BD-P and NaOH-P) constituted 8% in the 0–5 cm layer and 16% in the 5–10 cm layer. In both layers, the fraction with the lowest contribution was the iron-bound P fraction (BD-P), which was 1% and 2%, respectively. Bottom sediments in CZ were characterised by a relatively similar share of individual fractions. In both layers, the Res-P fraction was also dominant, with its share exceeding 43%. The HCl-P fraction (phosphorus with calcium) also had a significant share of 34 and 42%, respectively. The fraction with the smallest share was, as in the IZ, the BD-P fraction (0.5 and 1%), and the sum of fractions with the highest availability did not exceed 7%. In contrast, at the sampling station FZ, the fraction representing HCl-P, whose share varied between 64 and 69%, was predominant. The sum of the three most mobile fractions did not exceed 5%. Significantly different percentages of individual fractions of TP were found in sediments from PZ and MZ. In PZ, in the 0–5 cm layer, the fraction with the highest content was, like in FZ, the HCl-P fraction, reaching 38%. On the other hand, in the 5–10 cm layer, the Res-P fraction prevailed with 56%. The lowest content of BD-P in both layers at this station was 7% and 3%, respectively. The total content of the three most mobile fractions (NH_4_Cl-P, BD-P and NaOH-P) was higher than in FZ, reaching 18% in the 0–5 cm layer and 15% in the 5–10 cm layer. Moreover, the 0–5 cm layer had the highest NH_4_Cl-P fraction (18%) of all the sampling stations. Considering the content of particular fractions of TP in MZ, it was found that the HCl-P fraction had the highest share (43% and 54% in both layers, respectively). On the other hand, the BD-P fraction showed the lowest contribution (4% and 3%, respectively). The total content of the three most mobile fractions (NH_4_Cl-P, BD-P and NaOH-P) was the highest of all stations in Sztynorckie Lake (31% in the 0–5 cm layer and 19% in the 5–10 cm layer, Fig. 4).

The content of organic matter in the bottom sediments of Sztynorckie Lake was on average 19% and varied from 3.1% (FZ, in the 5–10 cm layer) to 33.3% (PZ, in the 0–5 cm layer). In the canals (IZ and CZ), on the other hand, the mean content of organic matter was slightly lower—16%. The minimum (10.2%) was noted in IZ in the 5–10 cm layer and the maximum of 22.1% in CZ in the 0–5 cm layer. On all analysed zones, the higher content of this parameter was found in the 0–5 cm layer (Table 4).

**Figure 5:**
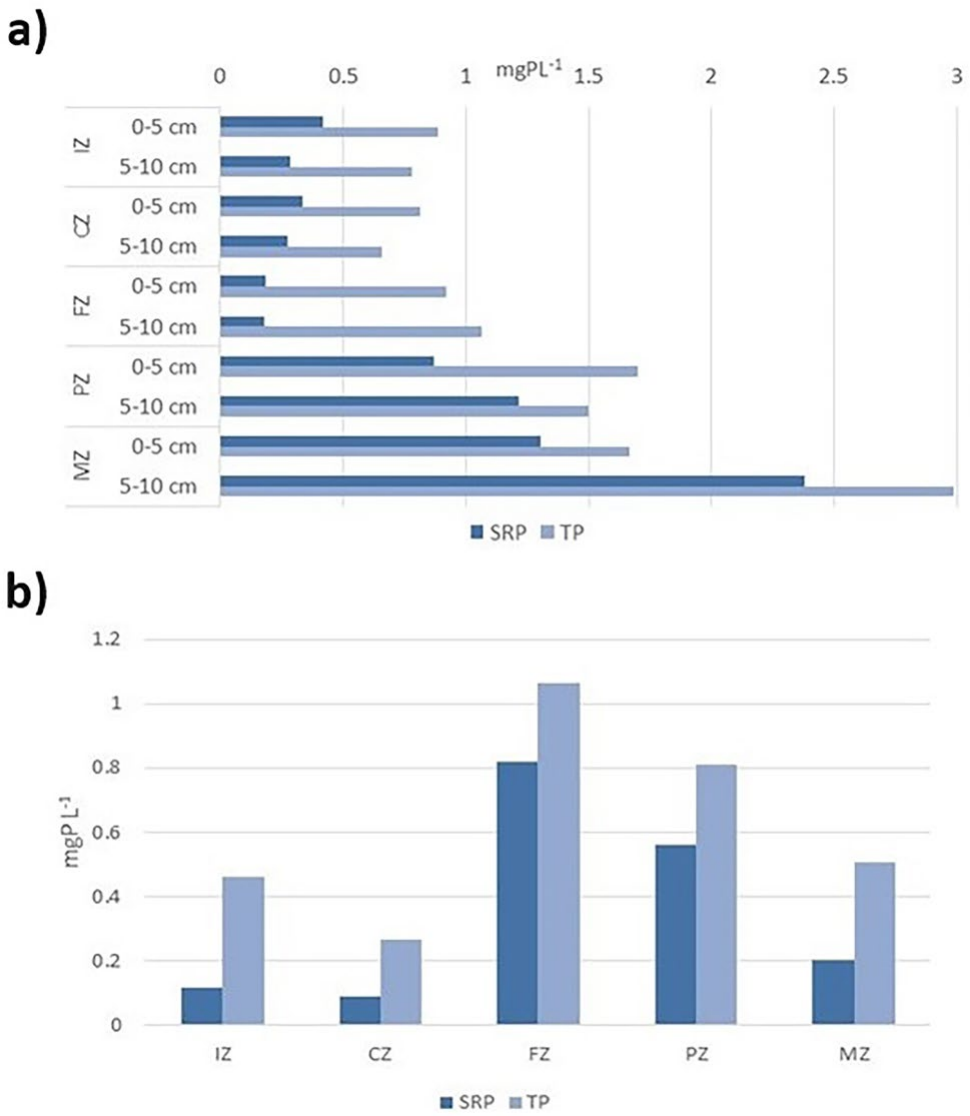
Soluble reactive phosphorus (SRP) and total phosphorus (TP) in interstitial water in bottom sediments (**a**) and overlying water (**b**) of Sztynorckie Lake in five zones [abbreviations—see Fig. 1].

In interstitial waters of bottom sediments, higher concentrations of both forms of phosphorus (SRP and TP) were found in PZ and in MZ, where in most cases they exceeded 1 mg P L^−1^. In the IZ and CZ zones, they were significantly lower and reached 0.89 mg P L^−1^ (Fig. 5a).

### Overlying water physicochemical parameters

SRP and TP concentrations in overlying water in Sztynorckie Lake varied over a wide range. Minimum values for both forms were noted in CZ—0.09 mgP L^−1^ and 0.27 mgP L^−1^, respectively, with maximum values in FZ—0.82 mgP L^−1^ and 1.06 mgP L^−1^. Sampling stations located directly in the lake basin (FZ, PZ and MZ) were characterised by higher concentrations of both phosphorus forms in the overlying water than in zones IZ and CZ, which did not exceed 0.5 mgP L^−1^ (Fig. 5b).

In PZ, from April to October, the bottom waters were characterised by a quite high temperature, which reached 20 °C, good oxygenation, which did not reach values lower than 5.3 mgO_2_L^−1^ and a high pH, which in fall reached 9.2.

## Discussion

The study showed that the shallow Sztynorckie Lake is characterised by different types of bottom sediments occurring in five distinguished zones. The intensive boating activity, recreational and agricultural use of the direct catchment area, transformation of the shoreline, and location of the marina and fuelling station above the lake not only affect the quality of the lake’s water but also the composition of bottom sediments and the possibility of internal phosphorus loading.

The sediments from the Profundal Zone of Sztynorckie Lake (station 4—PZ) were of dark-grey algal gyttja character and were characterised by the highest water content (above 90%) and the lowest dry residue content. Thus, this indicates a slight consolidation of the sediment structure and its liquid character. Such a type is characteristic of lake sediments and higher trophy, in which a high level of primary production mainly of phytoplankton is observed. Sediments from PZ were also characterised by the highest content of TP, resulting from the mineralisation of the organic matter originating from the settled phytoplankton^59^. The bottom sediments of the profundal were also characterised by the highest content of organic matter in the sediment, higher in its surface layer (0–5 cm) than deeper (5–10 cm). This situation resulted from increased sedimentation of autochthonous organic matter (phytoplankton) throughout the water column. Phytoplankton remnants belong to easy-decomposing organic matter particles, which influence higher phosphorus release from bottom sediments. Intensive aerobic and anaerobic decomposition processes can mobilise part of organic phosphorus during settling through the water column^60^.

One of the indicators determining the potential for phosphorus release from bottom sediments into the overlying water is the share of individual fractions of TP because they are characterised by different bioavailability and potential for release into water column^18,22^. The sediments of the profundal zone of Sztynorckie Lake were characterised by the highest total content of the most mobile fractions (NH_4_Cl-P, BD-P and NaOH-P) of all the studied sampling stations, which indicates a higher possibility of phosphorus release from the sediments of this zone in comparison with the others. The high mobility of phosphorus in the bottom sediments was also indicated by the high concentrations of both SRP and TP in the interstitial waters. Phosphorus concentration in pore waters is commonly regarded as an indicator of the intensity of its transport across the sediment–water interface and is the result of phosphorus turnover, namely sorption and mineralisation and phosphorus transport^61,62^. Higher concentrations of phosphorus in interstitial waters are observed in the pelagic than in the littoral zone^63^. In turn, rather high concentrations of both forms of phosphorus under consideration in the overlying waters may result from internal loading from bottom sediments. The phosphorus concentration in the interstitial and overlying water in lakes allows us to predict the phosphorus pool potentially available for release from bottom sediments^16,64^. The overlying waters of this zone are characterised by good oxygenation throughout the growing season, which ranges from 5.3 to 8.9 mgO_2_ L^−1^. Due to the low maximum depth (3.1 m), no oxygen deficits were found here. However, the release of phosphorus from the sediment occurs not only under anaerobic conditions^14,65,66^ but also in the presence of oxygen^60,65^. Well-oxidised sediment surface can be a sink of phosphorus (sorbing settling phosphorus), preventing phosphorus release from deeper sediments. Shallow lakes usually have extensive littoral areas, so the littoral sediments play an important role in phosphorus cycling^67,68^. The well-oxidised sediment surface does not preclude the potential release of mobile phosphorus to the overlying water due to gradients with high pore-water concentrations compared to low phosphorus concentrations in the overlying water^69^. Short-term changes in oxygen conditions are related to windless weather or high temperature, leading to periodic phosphorus release^70^. The good oxygenation of the overlying layer in the study lake is also because it undergoes mixing at the bottom.

The MZ was characterised by a relatively high content of TP in sediments. Similar to the PZ, the bottom sediments were characterised by rather high water (above 70%) and organic matter content. The organic matter most likely originated from sedimenting particles from the water column of autochthonous (primary production of phytoplankton and macrophytes in the littoral) and allochthonous origin (moored boats). Thus, this indicates a significant anthropogenic load on this part of the lake. A higher contribution of the most mobile fractions and a small share of the Res-P fraction, compared to others, indicated the significant potential for internal phosphorus loading from bottom sediments in this zone. In the exchange process, the contribution of the most mobile fractions is particularly important^71^. The highest share of the HCl-P fraction was found in both sediment layers, which can indicate macrophytic remains in sediments. The higher concentrations of dissolved phosphates and TP in the interstitial waters observed in this zone also indicated a possible release of phosphorus from the bottom sediments to the overlying waters^72^. Despite the good oxygenation of the overlying water in this zone of the lake, the release of phosphorus may be quite significant. Under oxic conditions, phosphorus release from organic matter mineralisation is controlled through the sorption capacity of sediments. Moreover, even with good oxygenation of the overlying water, anaerobic conditions often occur just below the sediment surface and the interstitial water has high phosphorus concentrations^65^. Resuspension of the bottom sediments of this lake may also contribute to increased internal supply. It significantly affects nutrient dynamics, especially phosphate at the sediment–water interface^73^. This process may be intensified due to the shallow depth of the reservoir and the intense mixing of waters by floating yachts and boats^7^. Wind-driven wave resuspension of sediments can cause rapid oxygen deficits in the overlying water layer, contributing to phosphate release^74^. Because of its shallow depth, Sztynorckie Lake is mixed at the bottom and is therefore very susceptible to the process of resuspension of bottom sediments.

The bottom sediments from the lake zone near FZ had different characteristics. In contrast to sediments from the PZ and MZ, they had the lowest water content (below 40%) and the highest dry residue content. However, they were characterised by the lowest amount of organic matter in the sediment. In this zone, developed recreational infrastructure, shoreline transformations due to concreting, lack of submerged macrophytes and a catchment with a high proportion of built-up areas were observed. Waves generated by boats can seriously increase shoreline erosion and indirectly affect submerged and emergent macrophytes. Plant communities are more susceptible to direct impacts from boat hulls or propellers^6^. The low potential for internal phosphorus supply in this zone was indicated by the lowest concentration of TP in the sediment compared to the other sampling stations. It was also confirmed by a low share of fractions with the highest bioavailability (NH_4_Cl-P, BD-P and NaOH-P) and a high phosphorus fraction occurring in practically insoluble mineral and organic compounds (Res-P)^16^. The Res-P fraction is practically useless in the phosphorus release from bottom sediments and is characterised by the lowest solubility and bioavailability and is permanently accumulated in bottom sediments^17^. Thus, the sediment of this zone does not represent a significant internal source of phosphorus, but the high mineral content (more than 95%) may indicate both intensive mineralisation of sedimentary matter and other anthropogenic contaminants originating from the boat-fuelling station located here. Fuel spills and emissions that contribute to the introduction of metals, hydrocarbons and other pollutants into water can be dangerous to the aquatic ecosystem^6^. Furthermore, the resuspension of bottom sediments intensified by passing boats could accelerate the mineralisation of organic matter^10,66^.

The bottom sediments of the two remaining zones, IZ and CZ, were of a slightly different composition than those discussed earlier. As for water content and dry residue, the sediments of these two zones were intermediate between FZ, PZ and MZ. The water content of these sediments was higher than in the FZ and lower than in the PZ and MZ. An inverse relationship was observed for the dry residue content. With organic matter, the concentrations found were slightly higher at the CZ than at the inflow, which resulted from an increased amount of organic matter originating from macrophytes inhabiting this zone. Organic matter present in sediments in this zone originated mainly from macrophytes and is not as easily degradable as fine matter originating from phytoplankton, observed in PZ^14,24^. A two-times higher concentration of TP in bottom sediments was observed in the IZ zone than in the CZ zone. This could result from the inflow of more phosphorus from the catchment in the case of the IZ zone, especially from agriculturally used areas^19,75^. The dominant fraction of phosphorus in both considered zones was the Res-P fraction, i.e. phosphorus practically unavailable biologically. The second largest fraction was the HCl-P fraction (phosphorus with calcium). HCl-P and Res-P fractions represent biologically inaccessible phosphorus^17^. However, the summed share of the fractions with the highest mobility was low. Such a share of particular fractions indicates low possibilities of these sediments in relation to internal phosphorus loading, which was confirmed by significantly lower phosphorus concentrations in interstitial and overlying waters compared to the other zones.

In lakes in areas of high tourist value, which are under significant recreational pressure, it is important to conserve/maintain good water quality. Very often, this is only possible through the combined introduction of protection measures in the catchment and restoration measures in the lake.

Sztynorckie Lake has controlled water and sewage management in the catchment area. Wastewater from the buildings located within the marina area is directed to the treatment plant in Nowy Sztynort, and the treated wastewater is discharged outside the lake’s catchment area. Protective measures in the catchment should concern two aspects: limiting the load of pollutants flowing with the waters of the tributary on the north-western side and maintaining or restoring a 100-m buffer zone around the lake to separate them from arable land. According to Izydorczyk^76^ the effectiveness of nitrogen and phosphorus removal from shallow groundwater through a buffer zone characterised by a mixed structure and plant species composition, with a width exceeding 30 m, may range from 60 to 90%. Pollutants in the tributary can be reduced through implementing the Hybrid Sequential Biofiltration System (HSBS) directly on the tributary. This system uses two parallel barriers: geochemical (filter beds) and biological (beds with macrophytic vegetation)^77^. Tests carried out on small wastewater treatment plants (treating up to 100 m^3^ of wastewater per day) confirmed the effectiveness of the system. Significant reductions in pollutant levels were found, with reductions at the HSBS outlet averaging 16% (up to a maximum of 93%) for TP, 15% (up to a maximum of 97%) for total nitrogen, and 21% reduction in polychlorinated biphenyls toxicity (PCB EQ—equivalency)^77^.

On considering the morphometric parameters of Sztynorckie Lake, water quality and the potential for phosphorus release from bottom sediments, it was concluded that the most effective method for improving water quality would be sediment removal combined with phosphorus inactivation. This method is among the most effective, due to the deepening of the lake basin, the reduction of the excessive extent of macrophytes and the radical reduction of ‘internal loading’^31^. If the lake is dredged significantly, we further reduce the amount of resuspension—the transport of sediment particles into the water tone due to water circulation, caused by wind and, as with Lake Sztynorckie, boat traffic. Another advantage is the possibility of reducing cyanobacteria by directly removing akinetes (cells in a resting state) from the bottom sediments.

Considering that the greatest threat is posed by the so-called modern sediments (about 150 years old), which contain 90% of all phosphorus in all components of the lake ecosystem, removing a minimum 10-cm layer is recommended. With Sztynorckie Lake, the sediment thickness is very high in the open water zone, confirmed by soundings conducted during sampling and by the fact that the present maximum depth is 2.8 m and not 3.1 m, as stated by the IFI^43^. Taking this into account, it is proposed to remove 0.5 m of sediment layer from the area delimited by the 2.5-m isobath (the area of the 2.5-m isobath is 117,645 m^2^, Online Appendix 1). In the littoral zone of the reservoir, the sediments are highly mineralised (only 5% organic matter), and their removal is not recommended. Additionally, the littoral zone acts as a natural barrier to protect the reservoir from the inflow of pollutants from the direct catchment. Only the removal of excess vegetation that poses a threat to the free movement of boats and water equipment is acceptable.

The phosphorus inactivation method is based on the precipitation of bioavailable phosphorus from the water column and its immobilisation in the bottom sediments by a coagulant. With Sztynorckie Lake, where the removal of bottom sediments is planned as the first stage of restoration, these measures are primarily aimed at removing particles of organic matter and algal cells from the water column. An additional aim is to increase the sorption capacity of the exposed bottom sediment layers so that they can act as a natural buffer for phosphorus loads from natural sources in the future. Iron, aluminium and calcium compounds are most commonly used as coagulants. Due to the very good oxygen conditions in Sztynorckie Lake, it is possible to use an environmentally safe iron coagulant. With iron compounds, the phosphorus precipitation mechanism is mainly based on the sorption of PO_4_^3−^ ions on the surface of the formed Fe(OH)_3_ flocs.

Chemical phosphorus binding as FePO_4_ plays a minor role. Given that iron compounds are most effective at a pH between 5 and 7 (the pH in Sztynorckie Lake fluctuates around 9) and reduce organic matter and suspended solids from the water-depth to a small extent, which is very important with Sztynorckie Lake, it is possible to use an additional coagulant based on aluminium compounds. Optimal conditions for applying aluminium salts occur at a water pH between 6 and 8. The predominant hydrolysis product is then the insoluble polymeric form—Al(OH)_3_. Immobilisation of phosphorus takes place through direct binding of inorganic phosphates to aluminium or through their adsorption onto the surface of the formed flocs.

The suggested combination of protective measures in the catchment area of Sztynorckie Lake and restoration measures in the reservoir itself, with balanced recreational pressure, should visibly improve water quality.

After the improvement in water quality (lake and inflow) has been achieved, the introduction of a ‘Protection area of inland water reservoir’ should be considered, which introduces many prohibitions, orders and restrictions within the direct catchment area aimed at improving and/or maintaining the good ecological status of the lake waters^78^.

## Conclusions

It can be concluded that the sediments of Sztynorckie Lake are differentiated in the identified zones, varying mainly in the intensity of recreational and catchment use. They have both different physicochemical properties and possibilities of phosphorus release to the overlying water. The greatest abilities of the internal supply of phosphorus are characteristic of the sediments in the PZ (where the boating activity, high organic matter sedimentation and mineralisation are), which is confirmed by the highest concentration of TP and the share of its individual fractions. Bottom sediments from the MZ (impact of boating activity, tourist service and port infrastructure) were characterised by similar parameters. The resuspension of sediments, intensified by boats, is also of great importance here. On the other hand, sediments in the FZ (with recreation infrastructure, transformation and development of the shoreline) have a low potential for phosphorus release but may be dangerous in terms of accumulated substances from fuels and other pollutants of anthropogenic origin. A slightly different character was found in sediments originating from zones connected with inflow (impact of agriculture’s catchment areas) and outflow from the lake (boating activity). Their composition was determined mainly by substances flowing from the agriculturally used catchment or from the littoral zone of the lake with macrophytes, and they have low possibilities of P release to overlying water. Thus, the sediments even in such a small and shallow lake, due to different anthropogenic pressures (recreation and catchment), varied in composition. Additionally, an analysis of individual TP fractions, a tool for assessing the internal source of phosphorus, showed that bottom sediments may also represent an additional source of phosphorus supply to this lake and contribute to further eutrophication of the ecosystem.

Taking into account the above-mentioned differences in bottom sediments, improving water quality in Sztynorckie Lake necessitates introducing, apart from protective measures in the catchment (preservation of the rushes zone, limiting nutrient loads flowing with inflow waters), restoration measures consisting of the removal of bottom sediments with the greatest potential for phosphorus release into the water-depth, i.e. within the profundal area. Protection and restoration measures planned in this way, while maintaining a sustainable recreational impact, should have the expected effect of improving water quality.

### Supplementary Information

Supplementary Figures.

## Data Availability

The datasets used and/or analysed during the current study available from the corresponding author on reasonable request.
