# Supplementary material for: Bottom sediments as an indicator of the restoration potential of lakes—a case study of a small, shallow lake under significant tourism pressure
Source: Sci Rep. 2024 Jun 11;14:13438. doi: 10.1038/s41598-024-64058-9 (PMC11166673; doi:10.1038/s41598-024-64058-9)
Supplement: Supplementary file 1 — Supplementary Figures. [file 41598_2024_64058_MOESM1_ESM.pdf]

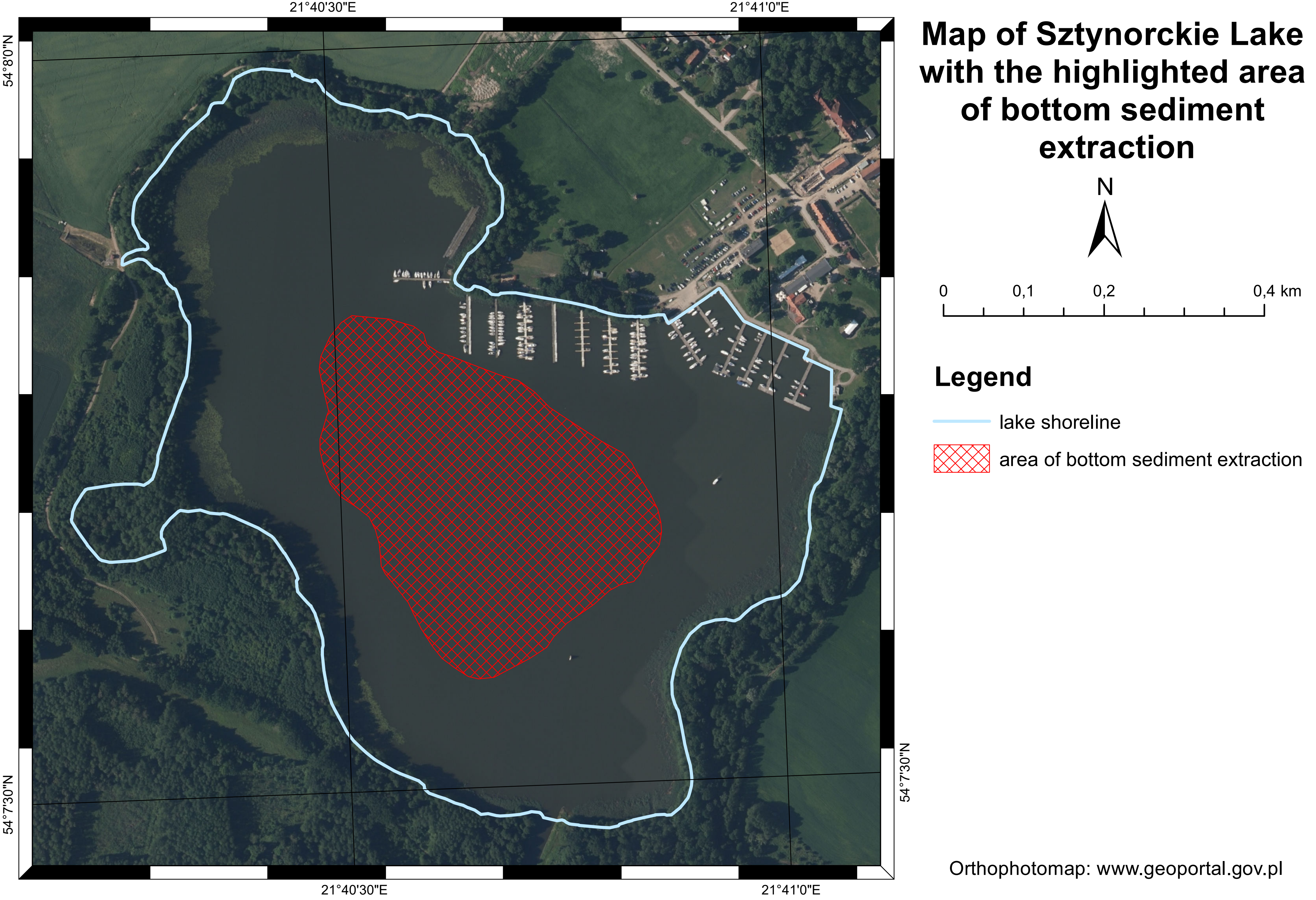
Map of Sztynorckie Lake with the highlighted area of bottom sediment extraction
N
0
0,1
0,2
0,4 km
Legend
lake shoreline
area of bottom sediment extraction
21°40'30"E
21°41'0"E
54°8'0"N
54°7'30"N
Orthophotomap: www.geoportal.gov.pl
